# Trends of Drain Placement During Revisional Bariatric Surgeries and Its Association with 30-Day Morbidity: An MBSAQIP Analysis of 64,495 Patients

**DOI:** 10.3390/jcm14072456

**Published:** 2025-04-03

**Authors:** Daniel Meyer, Sukhdeep Jatana, Daniel W. Birch, Noah J. Switzer, Shahzeer Karmali, Valentin Mocanu

**Affiliations:** 1 Department of Surgery, University of Alberta, Edmonton, AB T6G 2B7, Canada; dmeyer2@ualberta.ca (D.M.);; 2 Centre for Advancement of Surgical Education and Simulation (CASES), Royal Alexandra Hospital, Edmonton, AB T6G 2B7, Canada; 3 Digestive Disease & Surgery Institute, Cleveland Clinic, Cleveland, OH 44195, USA

**Keywords:** bariatric surgery, revisional surgery, conversional surgery, drain, postoperative complications

## Abstract

**Background:** Drains are often placed during bariatric procedures; however, their use in conversional or revisional bariatric surgery (CRBS) has not been thoroughly explored. Our study sought to identify the frequency of drain placement in CRBS, and characterize factors associated with drain placement and their influence on 30-day serious complications. **Methods:** Patients undergoing CRBS between 2020 and 2022 were included from the MBSAQIP database. Patients were placed into drain placed (DP) versus no drain (ND) cohorts and baseline characteristics and complication rate were compared. Multivariable logistic regression models were used to identify independent predictors of drain placement and complications. **Results:** of 64,495 included patients, drains were placed in 19.1% in 2020; this was down to 14.4% in 2022. Drain placement was associated with increased risk of multiple complications such as hemorrhage, readmission, surgical site infection, and gastrointestinal bleeding. On multivariate analysis, drain placement was an independent predictor of serious complications (aOR 1.45, *p* < 0.001), anastomotic leak (aOR 2.25, *p* < 0.001), organ space infection (aOR 2.12, *p* < 0.001), and reoperation (aOR 1.37, *p* < 0.001), as well as excess LOS (aOR 2.06, *p* < 0.001). Predictors of drain placement include older age, higher BMI, smoking status, history of venous thromboembolism, and procedural factors, such as undergoing non-sleeve revisional surgery or having an intraoperative leak test. **Conclusions:** Drain placement during CRBS surgical procedures is common and more likely in higher risk patients and anastomotic revisional procedures. Though the reasons for drain placement were not available, these data suggest that surgeons should be judicious in selecting patients for drain placement due to its association with increased LOS and postoperative morbidity in CRBS.

## Simple Summary

Bariatric surgery remains the most effective long-term solution for weight loss, and as its popularity rises, there are an increasing number of patients who may require revision of their previous procedure or conversion to another procedure for additional weight loss or to manage side effects of the surgery. These surgeries tend to be higher risk and drain placement may play a role in improving overall short-term complications by catching or mitigating the impact of complications. In our study, which assessed over 60,000 patients from MBSAQIP centers, drain placement was common but decreasing year by year. It was associated with higher risk of 30-day complications, even in context of surgery type, duration, and other patient factors. Anastomotic procedures and more comorbid patients were usually more likely to have drain placement. Though firm conclusions about the impact of drains on revisional or conversional bariatric surgery are difficult to make, given the lack of granular operative or intraoperative decision-making data, surgeons should be judicious when it comes to drain placement.

## 1. Introduction

Conversional or revisional bariatric surgery (CRBS) is commonly performed for patients with insufficient weight loss, weight recidivism, and gastroesophageal reflux disease (GERD), amongst other indications [1,2,3,4]. The postoperative CRBS patient is at risk of numerous complications and, of these complications, hemorrhage and gastrointestinal leak are major causes of readmission and reoperation [5,6,7,8]. Early detection and intervention result in reduced morbidity and mortality [9], prompting numerous strategies of mitigation and identification of these complications, including oversewing of staple lines [10,11], staple line reinforcement [11,12], topical sealants [13,14], intraoperative provocative leak testing [15,16], routine intraoperative drain placement [17,18,19], and routine postoperative imaging [17,20]. All such strategies have yielded mixed results with no clear indications for their routine. Both the American Society of Metabolic and Bariatric Surgeons (ASMBS) position statement published in 2015 [21] and The Enhanced Recovery After Surgery guidelines on bariatric surgery [22] suggest against routine drain placement in bariatric surgery; however, there is no clear indication or suggestion by guideline groups regarding drain placement in CRBS. Given the increased challenges and higher complication rates in CRBS compared to primary surgery, it is harder to generalize literature of primary surgery to CRBS. Thus, the role of drain placement in CRBS remains a gap in the literature.

Of these strategies, prophylactic drain placement is a common practice, with the most recent reports suggesting that up to 30% of patients undergoing metabolic and bariatric surgery have a drain placed at the time of their operation [23]. There are limited data that focus on the CRBS population which represents a large gap in the literature as CRBS patients represent a significantly different subset of patients due to the complex nature of repeat foregut/bariatric surgery. This practice has been under intense scrutiny, with a mounting body of literature demonstrating that routine drain placement is associated with increased risk of 30-day postoperative complications, reoperation, and death [23,24,25,26]. Despite these data, there is a significant proportion of patients with intraoperative drain placement, with limited high-quality prospective studies supporting their use or advocating against their utilization. Furthermore, it has not been clearly established who may benefit from drain placement, and if there are certain populations who are more likely to have a drain placed intraoperatively.

As a result, the primary outcomes of our study were to firstly characterize the frequency of intraoperative drain placement in CRBS cases, and, secondly, to characterize independent factors associated with drain utilization. Our secondary outcome was to determine if intraoperative drain placement had an influence on 30-day serious complications or length of stay after adjusting for comorbidities. In other words, how often are drains being left in CRBS, who are we placing them in, and what are the consequences, if any?

## 2. Materials and Methods

Data were extracted from the Metabolic and Bariatric Accreditation and Quality Improvement Program (MBSAQIP) registry. The MBSAQIP is the largest clinical dataset in North America and captures bariatric procedures across over 900 accredited bariatric centers. Contributing centers are subject to a rigorous independent accreditation process in accordance with internationally recognized bariatric surgical standards. Prospective data collection is performed by trained professional clinical reviewers and are subject to frequent reviews to ensure accuracy, with a target of data integrity audit disagreement rate of ≤5%. Ethics approval was not sought for this study given the nature of the MBSAQIP database, which is collected and stored anonymously. Detailed information about the reporting of outcomes, abstraction, or storage of data is available in the MBSAQIP Participant Use Data File [27]. As data were collected anonymously and stored in a secure database for patients of MBSAQIP centers, ethics approval was not sought for this study.

All patients undergoing CRBS laparoscopically between 2020 and 2022 were included in this retrospective cohort study. Conversion surgery was defined as conversion from one operation to another, while revision surgery was defined as revision of the same procedure. Patients undergoing primary surgery, emergency surgery, open surgery, or laparoscopic converted to open surgery were excluded. Open surgery or surgeries converted to open may be due to difficult operations that can lead to selection bias. In addition, emergency surgery presents a similar situation in which high-risk patients who are more prone to complications are more likely to have drain placement and was thus excluded. Patients were then divided into two cohorts: a no drain placed cohort (ND) and a drain placed cohort (DP), based on the presence or absence drain placement. The primary outcome was to characterize the frequency of drain placement and characterize independent factors that drive surgeons to leave drains. The secondary outcome was to determine if intraoperative drain placement had an influence on 30-day serious complications after adjusting for comorbidities.

Data collected included patient factors and operative factors. Patient factors included age, sex, body mass index (BMI), functional status (independent, partially dependent, and dependent prior to surgery), smoking status, the American Society of Anesthesiologists (ASA) physical status classification, and comorbidities including diabetes, hypertension, GERD, chronic obstructive pulmonary disease (COPD), chronic steroid use, renal insufficiency, dialysis, prior venous thromboembolism (VTE), therapeutic anticoagulation, oxygen dependence, sleep apnea, prior myocardial infarction, and prior cardiac surgery. Technical factors included the procedure type, presence or absence of intraoperative leak testing, drain placement, and operative length.

Specific postoperative complications collected included anastomotic leak, bleed, myocardial infarction, cerebrovascular accident, venous thromboembolism, pulmonary embolism, pneumonia, acute kidney injury, deep and superficial surgical site infections, wound disruption, sepsis, unplanned intubation, and a composite variable of overall serious complications. Additionally, length of stay (LOS), 30-day reoperation, intervention, readmission, and mortality rates were assessed. Excess LOS was defined as LOS greater than two standard deviations above the median, resulting in our definition being discharge from hospital between five and thirty days, inclusively, post operatively.

Categorical variables were expressed as absolute values and percentages, and univariate analysis was performed using Chi-squared tests. Continuous variables were expressed as weighted means ± standard deviations and analysis was performed using independent two sample *t*-tests.

To control for differences between groups and determine independent influence of intraoperative drain placement on 30-day serious complications, a non-parsimonious multivariable logistic regression model was developed using a hypothesis-driven purposeful selection methodology. A multivariate linear regression model was created to assess the independent influence of drains on LOS. Bivariate analysis of variables with a *p*-value < 0.1 or from variables previously deemed clinically relevant to our primary outcome were used to generate a preliminary main effects model. The variance inflation factor was calculated for each variable in each model. Values > 10 were deemed collinear and excluded from the model. Models were interrogated using the Brier score and the Receiver operating characteristic (ROC). Statistical analysis was completed using STATA 18 statistical software (StataCorp, College Station, TX, USA).

## 3. Results

### 3.1. Basic Demographics, Comorbidities, and Operative Characteristics

A total of 64,495 patients in the MBSAQIP registry underwent CRBS between 2020 and 2022 that met inclusion for our study. Of those, 10,573 (16.4%) had a drain placed (DP) and 53,922 (83.6%) did not (ND). We noted a downward trend (*p* < 0.001) in drain placement annually from 19.1% in 2020, to 16.4% in 2021, to 14.4% in 2022 (Table 1).

#### 3.1.1. Basic Demographics and Comorbidities

The mean age was 48.5 years, with the DP cohort being slightly older (48.9 ± 10.6 DP vs. 48.0 ± 10.6 ND, *p* < 0.001) and having a slightly higher BMI (41.6 ± 8.9 DP vs. 41.4 ± 8.2 ND, *p* < 0.001). The majority in both cohorts were female (87.3% DP vs. 88.0% ND) and had an independent functional status though the DP cohort was slightly more likely to be partially dependent or fully dependent (0.8% vs. 0.5%, *p* < 0.001). DP patients were also more likely to be smokers (5.5% vs. 4.9%, *p* = 0.005). ASA categorization was comparable between groups as most were ASA class II or III. In general, patients with drains had more comorbidities, such as hypertension (42.1% vs. 40.3%, *p* = 0.001), COPD (1.3% vs. 1.1%, *p* = 0.02), GERD (58.3% vs. 55.2%, *p* < 0.001), renal insufficiency (0.6% vs. 0.4%, *p* < 0.001), prior VTE (4.5% vs. 3.4%, *p* < 0.001), and sleep apnea (28.1% vs. 26.7%, *p* = 0.005), among others. A summary of patient demographics and comorbidities is presented in Table 1.

#### 3.1.2. Operative Characteristics

The most common procedure performed was RYGB revision/conversion comprising 68.1% of our sample (66.7% ND vs. 75.2% DP), followed by sleeve revision/conversion in 23.6% (25.4% ND vs. 14.1% DP), OAGB revision/conversion in 4.9% (6.3% DP vs. 4.6% ND), SADI revision/conversion in 2.8% (3.7% DP vs. 2.6% ND), and BPD-DS revision/conversion in 0.7% (0.7% in both DP and ND cohorts). This is also presented in Table 1.

Drains were placed most often in patients undergoing SADI revision/conversion (22.0%), followed by BPD-DS revision/conversion (21.1%), RYGB revision/conversion (18.1%), OAGB revision/conversion (17.2%), and sleeve revision/conversion (9.8%). Drain utilization differed significantly among procedure performed (*p* < 0.001).

Operative time was significantly longer in the DP cohort at 157.1 ± 87.8 min versus 133.7 ± 67.2 min in the ND cohort (*p* = 0.001).

Intraoperative leak testing was performed in 84.3% of patients (90.3% DP vs. 83.1% ND, *p* < 0.001). Information about the type of leak testing performed (e.g., endoscopic evaluation versus nasogastric instillation of methylene blue or other) is not available in our dataset, nor are post-operative imaging studies or duration of drain placement.

#### 3.1.3. Independent Risk Factors for Drain Placement

Multivariate logistic regression models demonstrated numerous independent risk factors leading surgeons to place drains, which are summarized in Table 2. These included demographic and patient factors such as increasing age (aOR 1.09, *p* < 0.001) and higher BMI (aOR 1.03, *p* < 0.001). Among comorbidities, only patients who were smokers (aOR 1.15, *p* = 0.010), had renal insufficiency (aOR 1.42, *p* = 0.029), or history of VTE (aOR 1.21, *p* = 0.001) had increased odds of drain placement. Among operative factors, operative length (per minute, aOR 1.00, *p* < 0.001) and the type of procedure both were independently associated with drain placement. When compared to SG revisions/conversion, those undergoing RYGB revision/conversion were more likely to have a drain placed (aOR 1.66, *p* < 0.001), as were OAGB revision/conversion (aOR 2.06, *p* < 0.001), BPD-DS revision/conversion (aOR 2.18, *p* < 0.001), and SADI revision/conversion (aOR 2.37, *p* < 0.001). The ROC AUC is 0.614 and BS 0.134, indicating good fit.

### 3.2. Post Operative Complications and Length of Stay

#### 3.2.1. Bivariate Analysis

Drain placement was associated with increased likelihood of 30-day postoperative complications which are summarized in Table 3. This included anastomotic leak (DP 1.7% vs. ND 0.7%, *p* < 0.001) and hemorrhage (DP 2.6% vs. ND 1.4%, *p* < 0.001), readmission (7.9% vs. 5.5%, *p* < 0.001), re-intervention (2.6% vs. 1.8%, *p* < 0.001), and reoperation (3.8% vs. 2.4, *p* < 0.001). The DP cohort also had significantly increased frequency of CVA, VTE, unplanned intubation, pneumonia, AKI, deep and superficial SSI, pulmonary embolism, renal failure, sepsis, GI bleeding, and bowel obstruction. There was no associated difference with wound disruption (*p* = 0.053), urinary tract infection (*p* = 0.182), and myocardial infarction (*p* = 0.706). Drain placement was associated with increased mortality on bivariate analysis (0.26% DP vs. 0.13% ND, *p* = 0.002) but not multivariate analysis, as discussed below.

#### 3.2.2. Multivariate Logistic Analysis of Complications

Multivariate logistic regression models revealed that drain placement was independently associated with increased odds of serious complications (aOR 1.45, *p* < 0.001) and had among one of highest predictive values (Table 4). The ROC AUC is 0.640 and BS 0.0588, indicating good fit. Drain placement was also significant in other multivariate models, including showing increased odds of anastomotic leak (aOR 2.25, *p* < 0.001), organ space infection (aOR 2.12, *p* < 0.001), and reoperation (aOR 1.37, *p* < 0.001). Drain placement was not independently associated with increased mortality (aOR 1.42, *p* = 0.146).

#### 3.2.3. LOS Trends and Comparisons Among DP and ND

LOS decreased across the study period with mean LOS 1.8 ± 2.1 (median 1, IQR 0–46) days in 2020, 1.7 ± 2.1 (median 1, IQR 0–53) days in 2021, and 1.6 ± 2.1 (median 1, IQR 0–48) in 2022 (Table 1). Excess LOS was significantly associated with drain placement (ND 2.6% vs. DP 6.7%, aOR 2.06, *p* < 0.001). Rate of excess LOS decreased throughout the study period with of excess LOS aOR 0.94 in 2021 and aOR 0.84 in 2022 (both compared to 2020). There was a marked increase in excess LOS in patients with dependent functional status (aOR 9.64, *p* = 0.001). Other factors significantly associated with prolonged LOS are summarized in Table 5 and included older age, presence of comorbidities including COPD, renal insufficiency, and history of VTE, as well as operative factors including operative length and non-sleeve revision/conversions. Higher albumin was associated with decreased likelihood of excess LOS (aOR 0.35, *p* < 0.001).

## 4. Discussion

We present the largest study examining the impact of intraoperative drain placement in CRBS populations using a large database registry with prospectively collected data. The primary questions this retrospective review sought to answer are as follows: how often are drains being left in revisional cases, who are we placing them in, and what are the consequences, if any?

To answer our first question, we found that drain utilization decreased over our study period. Among all patients undergoing metabolic and bariatric surgery in our series, we observed an overall decrease in drain use over time, with 19.1% of patients receiving a drain in 2020 dropping to 14.4% in 2022. Prior reports which primarily examined patients undergoing primary metabolic and bariatric surgery reported utilization as high as 57% [28] in 2006 to 2009, 36.4% in 2015, 33.6% in 2016, 30% in 2017 [23], and 24.5% in 2018 [29]. The reason for this decrease in drain placement is likely multifactorial owing to the fact that there is a body of literature in the bariatric surgical population as well as other subspecialties within surgery suggesting routine prophylactic drainage may be associated with anastomotic complications [28,29,30,31,32]. The primary literature does not seem to suggest utility in detecting bleeding [33,34] or leaks [18], or in the management of the latter [18]. Additionally, we suspect surgeons are becoming more comfortable with complex revisional procedures and are likely finding that drain utilization is not necessarily impacting clinical care or, in some cases, may prolong stays due to having to manage the drain.

To answer our second question, we can examine patient and operative factors. Our data demonstrate that surgeons are more likely to place drains in patients with increasing age and higher BMI. No specific comorbidity outside of a prior history of VTE, renal insufficiency and smoking was associated with drain placement. Such factors have been previous associated with morbidity and thus may lead to clinicians having a lower threshold for drain placement for these populations [35]. Operative factors included the duration of the procedure with longer procedures tending to have drains placed though the influence of drain placement on operation duration is unclear as drain placement may add more time or patients with longer procedures are selected for drain placement, due to possible difficulty or intraoperative concern. Drain placement also varied based on the type of procedure with patients undergoing SG revision/conversion being the least likely to have a drain placed, with placement in only about 9.8% of cases. Anastomotic procedures had significantly higher drain utilization rates, double that of SG, with utilization as high as 22.0% in SADI cases, 21.1% in BPD-DS, 18.1% in RYGB, and 17.2% in OAGB. This is likely owing to the increasing complexity of these procedures and the preponderance of surgeons to leave drains in order to monitor for anastomotic leak in these patients. SADI is the newest of the above procedures and is technically slightly easier than a traditional BPD-DS with one fewer anastomosis. Despite this, we found a slightly higher rate of drain placement, which may represent surgeons being cautious as they adopt this procedure into their practices.

In our study, drain placement was associated with a significantly increased risk for a serious complications, including hemorrhage, anastomotic leak, surgical site infection, organ space infection, readmission, reintervention, reoperation, and death.

Hemorrhage is a major intraoperative and postoperative complication following bariatric surgery [36] and numerous strategies to mitigate this, such as oversewing of staple lines [10,11], staple line reinforcement [11,12], and topical sealants [13,14] have been adopted with variable results in the literature supporting their use. Drains are utilized by some to allow for early detection of hemorrhage and prompt management. This question was specifically asked by Currò et al. who examined 100 SG patients with abdominal and nasogastric drainage and 100 patients without, finding that abdominal drainage does not prevent nor facilitate detection of bleeding or leaks after sleeve gastrectomy [33]. Other studies have identified drain placement as potentially increasing the risk of hemorrhage, including Gray et al. who found that drain placement was associated with an increased odds ratio of transfusion [26]. Dallal et al. also conducted a review of 352 patients who underwent laparoscopic RYGB without drain placement, finding six patients (1.7%) who had post-operative bleeding, all of whom were identified early by clinical examination using vital signs and physical examination [17]. This emphasizes the fundamental value of clinical assessment in the early post-operative period to detect complications in patients with obesity, namely that of tachycardia and/or hemodynamic instability to suggest hemorrhage [37,38].

Our study demonstrates that drain placement is associated with a significantly higher risk of anastomotic leak. Similar queries of the MBSAQIP database have also demonstrated increased risk of anastomotic leak, including Doumouras et al. who found that drain placement was associated with a 30% increased risk of leak among all patients undergoing SG or RYGB in 2015 [25]. Albanopoulos et al. also demonstrated that drain placement in patients undergoing SG was associated with an increased risk of leak (4.0% vs. 2.6%) as well as increased risk of general surgical complications [28]. Alizadeh et al. demonstrated that patients undergoing SG or RYGB had a higher risk of leak (1.6% vs. 0.4%) [29]. Of the most contemporary reports by Clapp et al., drain placement was associated with an increased risk of leak, readmission, reoperation, and death [23]. We agree with prior authors’ conclusions that this elevated risk of anastomotic leak and other complications are not likely a direct result of the drains themselves but rather represent a selection bias whereby surgeons are placing drains in patients they feel are high risk, though a randomized controlled trial would be necessary to determine this. Again, clinical assessment remains paramount for the detection of leak with mounting evidence that drains do not facilitate early detection of leak [33] and nor does routine post-operative imaging [17].

Other complications such as surgical site infection, organ space infection, readmission, reintervention, reoperation, and death have also been associated with drain placement in the literature and are consistent with our findings [23,26,39].

Excess LOS was independently associated with drain placement. Patients without drains were more likely to be discharged on post-operative day one to four (97.4% ND vs. 93.3% DP) and patients who were discharged between five to thirty days post operatively were over twice as likely to have had a drain placed at the time of their procedure (2.6% vs. 6.7%, aOR 2.06). We also noted that LOS decreased annually in the study period and, while there are several factors that play a part into this, we did note that it corresponded with an observed reduction in drain use over this same period. Other studies have found a similar association between drains and increased LOS leading to most contemporary enhanced recovery after surgery protocols to discourage routine drain placement [22,23,25,40,41].

The findings of this MBSAQIP study do not support routine placement of drains in CRBS. This is due to its association with longer operative times, longer lengths of stay, and increased risk of complications, which may be secondary to detection of clinically insignificant leaks or bleed. This echoes recent findings of a MBSAQIP paper assessing drain placement in primary bariatric surgery which showed similar results [39]. Most literature currently describes and assesses drain placement in primary surgery, and hence there may be limited generalizability to CRBS which is associated with increased risk. One of the author’s centers does not use drains during CRBS, while another author’s centers routinely place intraoperative drains during complex CRBS. A leak test is performed prior to drain removal. Populations that may benefit the most are those who may be at highest risk of leak, including those with lower albumin, history of VTE, longer operative length, and a RYGB conversion/revision (compared to SG conversion/revision). Current findings do not supersede clinical judgement, nor do the data take into account granular details of perioperative decision-making. The role of drain placement should be assessed on a case-to-case basis. Without the reason for drain placement, this study is limited on its ability to comment on its association with complications. Future studies should contain more granular information about the reason for drain placement (e.g., if it was routine, or due to concern of tissue quality or bleeding) and possibly considered as a MBSAQIP variable.

This study has several limitations based on the nature of its design. The most important one to highlight is that we do not have nuanced intraoperative details that may affect a surgeon’s choice to leave a drain, nor do we have the reason for drain placement. Furthermore, center-specific codes are also not available as some centers may routinely place drains while others may do so more selectively; such information could also influence interpretation of results. This leads to a selection bias that likely cannot be accounted for, even with multivariable modelling. Thus, associations with complications are noted but cannot be solely explained by drain placement. Additionally, we were only able to extract data using the MBSAQIP database’s pre-defined set of variables, which does not capture other nuanced data such as technical details about the procedures performed, including types of drains used (e.g., open or closed, active or passive), duration that drains were left in place, and postoperative radiographic leak testing. Improper usage of drains, such as those left longer than required, could also negatively affect the outcomes noted. The lack of details regarding drain management prevents such conclusions from being drawn. We also have no information about whether drains placed had any influence on the detection of anastomotic leak or altered the timeframe of their treatment. Furthermore, long-term complication data were not available from the MBSAQIP database and could not be assessed. In addition, due to the inclusion of only MBSAQIP centers in this study, which are North American, the results of this study may lack generalizability to the broader metabolic and bariatric surgery community.

Despite these limitations, we present the largest study to date investigating the use of abdominal drainage in revisional metabolic and bariatric surgical procedures. Though this study was not able to assess reasons for drain placement, the results suggest that drain utilization is associated with increased risk of serious complications and these findings should be considered intraoperatively prior to placement of a drain. Future studies should consider a randomized controlled trial design, with a decision to place a drain made at end of the case to avoid bias and to eliminate selection bias.

## 5. Conclusions

Drain placement during revisional bariatric surgical procedures is common but decreasing in frequency. Surgeons should be judicious in selecting patients for drain placement due to its association with excess length of stay and serious complications, though this would require future controlled studies to better ascertain the effect.

## Figures and Tables

**Table 1:** Baseline characteristics of patients undergoing revisional bariatric and metabolic surgical procedures, stratified by drain placement.

	No Drain *n* (% Total) *n* = 53,922	Drain *n* (% Total) *n* = 10,573	*p*-Value
Age at revisional/conversional surgery			<0.001
18–30	1677 (3.1%)	257 (2.4%)	
30–40	10,470 (19.4%)	1835 (17.4%)	
40–50	18,182 (33.7%)	3504 (33.1%)	
50–60	15,488 (28.7%)	3180 (28.9%)	
>60	8105 (15.0%)	1797 (15.4%)	
Mean	48.0 ± 10.6	48.9 ± 10.6	
Sex			0.072
Male	6487 (12.0%)	1338 (12.7%)	
Female	47,435 (88.0%)	9235 (87.3%)	
BMI			<0.001
<35	10,671 (19.8%)	2154 (20.4%)	
35–40	14,366 (26.6%)	2724 (25.8%)	
40–50	13,568 (25.2%)	2539 (24.0%)	
50–60	7993 (14.8%)	1504 (14.2%)	
60–70	5995 (11.1%)	1298(12.3%)	
>70	1346 (2.5%)	352 (3.3%)	
Mean	41.4 ± 8.2	41.6 ± 8.9	
Functional status			0.001
Independent	53,602 (99.5%)	10,481 (99.2%)	
Partially dependent	252 (0.5%)	79 (0.8%)	
Dependent	16 (0.0%)	12 (0.0%)	
ASA category			0.099
ASA I	106 (0.2%)	17 (0.2%)	
ASA II	12,536 (23.3%)	2395 (22.7%)	
ASA III	39,720 (73.7%)	7881 (74.5%)	
ASA IV	11,495 (2.8%)	263 (2.5%)	
ASA V	2 (0.0%)	2 (0.02%)	
Not assigned	63 (0.1%)	15 (0.1%)	
Smoker	2490 (4.9%)	520 (5.5%)	0.005
Diabetes			0.026
Non-diabetic & diet controlled	45,976 (85.3%)	8970 (84.8%)	
Non-insulin dependent	2004 (3.7%)	451 (4.3%)	
Insulin dependent	5942 (11.0%)	1152 (10.9%)	
Serum albumin (g/dL)	4.07 ± 0.4	4.05 ± 0.4	0.235
HTN	21,731 (40.3%)	4453 (42.1%)	0.001
COPD	579 (1.1%)	141 (1.3%)	0.020
DLD	10,839 (20.1%)	2267 (21.4%)	0.002
GERD	29,745 (55.2%)	6168 (58.3%)	<0.001
Renal insufficiency	199 (0.4%)	65 (0.6%)	<0.001
Dialysis	79 (0.2%)	24 (0.2%)	0.058
Prior VTE	1836 (3.4%)	472 (4.5%)	<0.001
Venous stasis	310 (0.6%)	71 (0.7%)	0.236
Therapeutic anticoagulation	1777 (3.3%)	409 (3.9%)	0.003
Sleep Apnea	14,432 (26.7%)	2969 (28.1%)	0.005
Prior MI	548 (1.0%)	111 (1.1%)	0.754
Prior PCI/PTCA	733 (1.4%)	176 (1.7%)	0.015
Prior cardiac surgery	444 (0.8%)	129 (1.2%)	<0.001
Immunosuppressant use	1476 (2.7%)	309 (2.9%)	0.288
Operative year			<0.001
2020	14,340 (80.9%)	3384 (19.1%)	
2021	19,063 (83.6%)	3738 (16.4%)	
2022	20,519 (85.6%)	3451 (14.4%)	
All	53,922 (83.6%)	10,573 (16.4%)	
Procedure			<0.001
SG revision/conversion	13,719 (90.2%)	1491 (9.8%)	
RYGB revision/conversion	35,974 (81.9%)	7953 (18.1%)	
BPD-DS revision/conversion	2479 (78.9%)	662 (21.1%)	
OAGB revision/conversion	365 (82.8%)	76 (17.2%)	
SADI revision/conversion	1385 (78.0%)	391 (22.0%)	
Intraoperative leak test	44,148 (83.1%)	9458 (90.3%)	<0.001
Operative duration (min)	133.7 ± 67.2	157.1 ± 87.8	0.001
Length of stay			<0.001
1–4 days	50,276 (97.4%)	9680 (93.3%)	
5–30 days	1344 (2.6%)	695 (6.7%)	

Abbreviations: ASA—American Society of Anesthesiologists; BMI—body mass index; COPD—chronic obstructive pulmonary disease; DLD—dyslipidemia; GERD—gastroesophageal reflux disease; HTN—hypertension; MI—myocardial infarction; PCI—percutaneous coronary intervention; PTCA—percutaneous transluminal coronary intervention; RYGB—roux-en-Y gastric bypass; VTE—venous thromboembolism; BPD-DS—biliopancreatic diversion with duodenal switch; OAGB—one anastomosis gastric bypass; SADI—single anastomosis duodenoileostomy; SG—sleeve gastrectomy.

**Table 2:** Predictors of drain placement of patients undergoing drain placement at time of revisional bariatric and metabolic surgical procedure.

Predictors of Drain Placement	aOR	95% CI	*p*-Value
Patient Factors			
Older age (per 10 years)	1.09	1.06–1.11	<0.001
Higher BMI (per 5 kg/m^2^)	1.03	1.01–1.04	<0.001
Female	0.95	0.88–1.02	0.160
Smoker	1.15	1.03–1.28	0.010
Functional status (vs. independent)			
Partially dependent	1.26	0.94–1.70	0.128
Fully dependent	0.80	0.17–3.67	0.769
Comorbidities			
Diabetes (vs. non-diabetic or diet-controlled)			
Non-insulin dependent	0.93	0.86–1.01	0.091
Insulin dependent	1.08	0.95–1.22	0.222
COPD	0.96	0.77–1.19	0.694
HTN	1.03	0.97–1.08	0.329
GERD	0.97	0.92–1.02	0.299
DLD	1.00	0.94–1.07	0.888
Renal insufficiency	1.42	1.04–1.94	0.029
History of VTE	1.21	1.08–1.36	0.001
Albumin	0.97	0.92–1.03	0.349
Surgical Factors			
Operative length (per min)	1.00	1.00–1.00	<0.001
Procedure (vs revision/conversion of SG)			
RYGB revision/conversion	1.66	1.54–1.78	<0.001
BPD-DS revision/conversion	2.18	1.94–2.45	<0.001
OAGB revision/conversion	2.06	1.55–2.74	<0.001
SADI revision/conversion	2.37	2.06–2.72	<0.001
Intraoperative leak test	1.57	1.45–1.70	<0.001

Abbreviations: aOR—adjusted odds ratio; BMI—body mass index; BPD-DS—biliopancreatic diversion with duodenal switch; CI—confidence interval; COPD—chronic obstructive pulmonary; DLD—dyslipidemia; GERD—gastroesophageal reflux; HTN—hypertension; OAGB—one anastomosis gastric bypass; RYGB—roux-en-Y gastric bypass; SG—sleeve gastrectomy; SADI—single anastomosis duodenoileostomy; VTE—venous thromboembolism.

**Table 3:** Post-operative complications of patients with drain placement at the time of revisional bariatric and metabolic surgical procedure.

Complication	No Drain *n* (%) *n* = 53,922	Drain *n* (%) *n* = 35,911	*p*-Value
Anastomotic leak	362 (0.7%)	177 (1.7%)	<0.001
Bleed	765 (1.4%)	278 (2.6%)	<0.001
Reoperation	1308 (2.4%)	406 (3.8%)	<0.001
Intervention	971 (1.8%)	279 (2.6%)	<0.001
Readmission	2981 (5.5%)	837 (7.9%)	<0.001
Death	69 (0.1%)	27 (0.3%)	0.002
Pneumonia	200 (0.4%)	81 (0.8%)	<0.001
AKI	56 (0.1%)	26 (0.3%)	<0.001
Deep SSI	558 (1.0%)	261 (2.5%)	<0.001
Wound disruption	42 (0.1%)	16 (0.1%)	0.053
Sepsis	157 (0.3%)	62 (0.6%)	<0.001
Unplanned intubation	82 (0.2%)	43 (0.4%)	<0.001
CVA	9 (0.0%)	7 (0.1%)	0.003
VTE	203 (0.4%)	62 (0.6%)	0.002
Serious complication	3030 (5.6%)	987 (9.3%)	<0.001
Superficial incisional SSI	370 (0.7%)	143 (1.4%)	<0.001
Postoperative deep incisional SSI	90 (0.2%)	37 (0.4%)	<0.001
Pulmonary embolism	91 (0.2%)	34 (0.3%)	0.001
Acute renal failure	28 (0.1%)	17 (0.2%)	<0.001
Urinary tract infection	216 (0.4%)	52 (0.5%)	0.182
Myocardial infarction	12 (0.0%)	3 (0.0%)	0.706
Postoperative sepsis	157 (0.3%)	62 (0.6%)	<0.001
Gastrointestinal bleed	310 (0.6%)	85 (0.8%)	0.019
Bowel obstruction	499 (0.9%)	132 (1.3%)	0.007

Abbreviations: AKI—acute kidney injury; CVA—cerebrovascular accident; SSI—surgical site infection; VTE—venous thromboembolism.

**Table 4:** Predictors of serious complications of patients undergoing drain placement at time of revisional bariatric and metabolic surgical procedure.

Predictors of Serious Complications	aOR	95% CI	*p*-Value
Patient Factors			
Older age (per 10 years)	1.04	1.00–1.08	0.043
Higher BMI (per 5 kg/m^2^)	0.90	0.87–0.92	<0.001
Race (vs. white)			
Black	1.24	1.14–1.35	<0.001
Other	0.96	0.85–1.08	0.501
Smoker	1.07	0.91–1.26	0.409
Functional status (vs. independent)			
Partially dependent	0.90	0.56–1.42	0.640
Dependent	0.85	0.11–6.64	0.877
ASA class (vs. class I and II)			
ASA class III	1.08	0.99–1.19	0.085
ASA class IV and V	1.41	1.13–1.76	0.003
Comorbidities			
COPD	1.37	1.05–1.80	0.021
HTN	1.10	1.01–1.19	0.029
GERD	1.17	1.08–1.27	<0.001
DLD	1.08	0.98–1.19	0.108
Renal insufficiency	0.98	0.61–1.55	0.917
History of VTE	1.95	1.69–2.25	<0.001
History of MI	1.51	1.15–1.98	0.003
Albumin	0.74	0.68–0.80	<0.001
Immunosuppressive therapy	1.14	0.93–1.38	0.198
Surgical Factors			
Drain placed	1.45	1.33–1.58	<0.001
Operative length (per min)	1.00	1.00–1.00	<0.001
Procedure (vs. revision/conversion of SG)			
RYGB revision/conversion	1.23	1.11–1.37	<0.001
BPD-DS revision/conversion	1.59	1.33–1.89	<0.001
OAGB revision/conversion	1.65	1.10–2.49	0.016
SADI revision/conversion	1.22	0.96–1.54	0.100

Abbreviations: aOR—adjusted odds ratio; ASA—American Society of Anesthesiologists; BMI—body mass index; BPD-DS—biliopancreatic diversion with duodenal switch; CI—confidence interval; COPD—chronic obstructive pulmonary; DLD—dyslipidemia; GERD—gastroesophageal reflux; HTN—hypertension; MI—myocardial infarction; OAGB—one anastomosis gastric bypass; RYGB—roux-en-Y gastric bypass; SG—sleeve gastrectomy; SADI—single anastomosis duodenoileostomy; VTE—venous thromboembolism.

**Table 5:** Predictors of excess length of stay in patients undergoing drain placement at time of revisional bariatric and metabolic surgical procedure.

Predictors of Serious Complications	aOR	95% CI	*p*-Value
Patient Factors			
Older age (per 10 years)	1.07	1.01–1.12	<0.017
Higher BMI (per 5 kg/m^2^)	0.79	0.77–0.82	<0.001
Race (vs. white)			
Black	1.3	1.15–1.47	<0.001
Other	1.19	1.00–1.42	0.044
Functional status (vs. independent)			
Partially dependent	2.13	1.34–3.40	0.002
Dependent	9.64	2.53–36.78	0.001
Comorbidities			
COPD	2.09	1.53–2.87	<0.001
HTN	1.06	0.95–1.20	0.302
GERD	0.89	0.80–0.99	0.049
DLD	1.04	0.91–1.19	0.567
Renal insufficiency	1.79	1.12–2.88	0.015
History of VTE	2.06	1.70–2.50	<0.001
History of MI	0.78	0.50–1.24	0.299
Albumin	0.35	0.31–0.39	<0.001
Surgical Factors			
Drain placed	2.06	1.84–2.31	<0.001
Operative length (per min)	1.01	1.01–1.01	<0.001
Procedure (vs. revision/conversion of SG)			
RYGB revision/conversion	1.74	1.45–2.09	<0.001
BPD-DS revision/conversion	2.13	1.63–2.79	<0.001
OAGB revision/conversion	2.11	1.14–3.9	0.018
SADI revision/conversion	1.98	1.42–2.8	<0.001

Abbreviations: aOR—adjusted odds ratio; BMI—body mass index; BPD-DS—biliopancreatic diversion with duodenal switch; CI—confidence interval; COPD—chronic obstructive pulmonary; DLD—dyslipidemia; GERD—gastroesophageal reflux; HTN—hypertension; MI—myocardial infarction; OAGB—one anastomosis gastric bypass; RYGB—roux-en-Y gastric bypass; SG—sleeve gastrectomy; SADI—single anastomosis duodenoileostomy; VTE—venous thromboembolism.

## Data Availability

The MBSAQIP database is accessible to MBSAQIP-designated centers.
